# Impact of *Enterococcus faecium* AL41 on growth performance, immune parameters, morphology, and tight junction proteins in intestine of chicks

**DOI:** 10.1016/j.psj.2025.105361

**Published:** 2025-05-28

**Authors:** Levkut Martin, Karaffová Viera, Grešáková Ľubomíra, Čechová Michaela, Faixová Zita, Szabóová Renáta, Rudolf Žitňan, Róbert Herich

**Affiliations:** a Department of Morphological Disciplines, University of Veterinary Medicine and Pharmacy in Košice, Slovakia; b Department of Biology and Physiology, University of Veterinary Medicine, Košice, Slovakia; c National Agriculture and Food Centre Research Institute for Animal Production, Nitra, Slovakia; d Institute of Animal Physiology Centre of Biosciences of the Slovak, Academy of Sciences, Šoltésovej 4, 040 01 Košice, Slovakia

**Keywords:** Growth performance, Intestinal immunity, Tight junction, Morphometry, Broiler

## Abstract

In the study we investigated how post-hatch preventive administration of *Enterococcus faecium* AL41 affects growth performance, the intestinal immune parameters and barrier factors, and morphology of jejunum in chickens. Chicks were divided into two groups as control (C) and *Enterococcus faecium* AL41 (EF). Samples (*n=10*) from blood and small intestine were taken at days 5, 8, and 11 of the life. Body weight gain increased in EF treated chicks on day 11. From day 8 to 11quantity of IgA in intestinal flush was lower in EF group. Relative expression of occludin gene was up-regulated at day11 in EF group and in same group claudin 1 gene was up-regulated at days 5 and 11. Jejunal CD3^+^*lamina propria* lymphocytes (LPL) showed stimulation at days 5 and 11. Similarly, CD4^+^, CD8^+^LPL cells were increased at day 8 in experimental group. IgA cells were stimulated at days 8 and 11in EF group. Finally, CD45^+^ LPL cells showed increase at day 11 of life in EF group. Morphological parameters of jejunum were increased in EF group as height of villi at day 1, cutting surface at days 5, 11 and depth of crypts at days 8 and 11. On the other hand, adding of *E. faecium* AL41to broilers did not show any impact on white blood cell counts in peripheral blood, phagocytic and metabolic activity, quantity of MUC-2, and intraepithelial lymphocytes (IEL). Our trial indicated that early administration of EFAL41after hatching affects growth performance improves health function as intestinal morphology, barrier integrity of gut and intestinal immunity.

## Introduction

A functional gut barrier composed of mucus and epithelial cells with tight junctions (TJs) is essential for optimal health and efficient production in poultry (Proszkowiec-Weglarz et al., 2020). Probiotics are used to regulate intestinal microbiota and can directly influence gut immune system through the pattern-recognition receptors (PRRs) present in both epithelial and immune cells of the host (Tarradas et al., 2020).

Modifications in gut microbiota of chickens induced by probiotics, prebiotics, and synbiotics have been demonstrated to improve conditions for the development of beneficial microbiota and suppressing those of pathogenic bacteria (Revajová et al., 2022). Probiotics are cultures of potentially beneficial bacteria that positively affect the host by regulating the microbial balance and restoring the normal intestinal permeability and gut micro-ecology (Ghareeb et al., 2012).

Intestinal mucus plays important roles in protecting the epithelial surfaces against pathogens. Mucus layer support the colonization with commensal bacteria, maintain an appropriate environment for digestion and facilitate nutrient transport from the lumen to the epithelial covering (Duangnumsawang, 2021).

The intestinal epithelium is important defence line against foreign antigens, such as toxins and pathogens from the gut lumen (Suzuki, 2013). The strongest protein complexes, known as tight junctions (TJs) join the apical side of intestinal epithelial cells (Cai et al., 2024). Tight junctions are one mode of cell-to-cell adhesion, and play a central role in sealing the intercellular space in epithelial and endothelial cellular sheets (Anderson and Van Itallie, 2009). Tight junctions regulate the passage of ions and molecules through the paracellular pathway (Tomita et al., 2004). Some enteric pathogens can induce permeability defects in gut epithelia by altering TJ proteins, mediated by their toxins (Awad et al., 2017). Claudin and occludin are major transmembrane proteins, that are often targeted and misplaced by viruses (Liu et al., 2009), bacteria (Lapointe et al., 2010), and inflammatory cytokines (Kirschner et al., 2009). Moreover, Beeman et al. (2012) suggest that occludin has signaling properties that initiate cell death when its tight junction location is disrupted.

Changes in microbiota are most likely the consequence of an altered condition of the intestinal wall. Growth promoting effect of probiotic organisms in animals is inconsistent in spite of positive influence on animal health (Niewold, 2007). Several papers (Awad et al., 2009; Levkut et al., 2020) reported that synbiotic product has beneficial effects on broiler performance parameters including average daily body weight gain (BWG) and feed conversion ratio (FCR).

Feeding *Enterococcus faecium* to broilers have been shown to reduce proliferation of pathogens, modify gut microbiota, minimize gut inflammation and modify mucosal morphology (Levkut et al., 2009; Žitňan et al., 2019; Revajová et al., 2022). However, in addition to earlier mentioned parameters, there is no experimental work on the effect of oral probiotic on the growth performance, intestinal morphology, intestinal barrier integrity and intestinal immunity early after hatching.

## Material and method

### Ethical approval

The chicks were handled and killed according to the Slovak state regulations. The experiment was approved by the Ethics Committee of the University of Veterinary Medicine and Pharmacy, Košice, Slovakia, and by the Committee for Animal Welfare of the Ministry of Agriculture of the Slovak Republic (permission number 1184-3/2020-220).

### Experimental design

Eighty one-day-old male COBB 500 chicks, obtained from a commercial hatchery (Baromfi-Coop Kft., Petneháza, Hungary), were randomly allocated into two experimental groups: a control group (C) and a treatment group (EF), each consisting of two replicates of 10 birds

Birds of EF group were inoculated with the probiotic strain *Enterococcus faecium* (*E. faecium*) AL41 (CCM 8558) as described by Karaffová et al. (2017). The probiotic strain *E. faecium* AL41 (EF) was grown as described by Letnická et al. (2017). From the first to the seventh day of the experiment, a suspension with 10^9^ colony-forming units of EF in 0.2 mL PBS was applied to every chicken of the EF group *per os*. To simulate the same stress to chicken of the C group, an equal volume of PBS only was applied with a Pasteur pipette. The chicks were placed in 4 pens (each pen represented one replicate) with an area of 2.06 m^2^ (length 165 cm, width 125 cm, height 120 cm) covered with wood shavings and fed a standard BR-1 (Čaňa, Košice, Slovakia), with access to water *ad libitum*. The composition of feed is shown in Table 1. All chicks were kept under similar conditions of management throughout the experiment in accordance with the broiler management guidelines (Cobb Broiler Management Guide, 2021). On the first day of placement, chicks were provided with 24 h of light. Then the light regimen of 23 h light /1 h dark cycle was maintained until day 6 of life, followed by a continuous 6 h dark period. Relative humidity varied from 60 to 70 % and the temperature regimen was adjusted to the particular age of the chicks according to breeding recommendations. The control of the temperature and humidity in the room was performed 8 times a day (every 3 h) with a KlimaLogg Pro monitoring device with a signalling system. Prior to the start of the experiment, faecal control samples were taken from the chicks for microbiological examination. The blood samples and the samples from all individual parts of the small intestine (5th, 8th and 11th day of the experiment) were collected from 10 chicks from both groups in each sampling.

**Table 1:** Composition of feed mixtures.

**Components**	**Starter D1-D11**
Corn % Soya extracted scrap % Wheat % Full-fat soya % Fodder lime % Monocalcium phosphate % Plant oil % Premix % Methionine % Lysine % Sodium bicarbonate % Threonine % Salt % Lupro-Cid nal % FRA LeciMax dry % l valine % Anticoccidials Myco fix select % **Declared values** Dry mass % ns % Fatt % Dietary fiber % Ash % MEn (mj.kg) Lysine % Methionine % Met+ lys % Threonine % Tryptophan % Valine % Ca % P total % Sodium % Mg % Zn (mg/kg)	42.77 25.0 20.0 7.0 1.21 1.17 0.6 0.5 0.36 0.30 0.25 0.16 0.16 0.30 0.05 0.05 Maxiban G160 50 mg/kg 0.08 87.83 20.33 4.09 2.65 5.46 12.53 1.27 0.64 0.99 0.88 0.23 0.95 0.79 0.65 0.15 0.14 125.27

ME_n_ is calculated value.

Vitamin and mineral premix: vitamin A 12,500 IU/kg, vitamin D3 4,000 IU/kg,.

vitamin E 80.00 mg/kg, Cu 15.00 mg/kg, vitamin D/25 cholekalciferol 1,000 IU/kg,.

Jod 1.00 mg/kg, Mn 50.00 mg/kg, Zn 90.00 mg/kg, Fe 40.00 mg/kg, Se 30.00 mg/kg.

For the current experiment, 10 birds *per* group were slaughtered at the beginning of the trial (one day chicks). Subsequently, randomly selected 10 birds *per* group (5 birds *per* replicate) were slaughtered at days 5, 8, and 11 of life. Samples from four randomly selected animals per group were immediately placed in RNA Later solution (Qiagen, Manchester, UK) for gene expression analyses and stored at −70°C until further processing.

The live body weight of each bird was recorded at the beginning of the trial and on days 8 and 11 of sampling. Average daily feed intake (ADFI) was recorded at the pen level as the difference between the feed offered and the feed refused. These data were used to calculate body weight gain (BWG), average daily weight gain (ADG) and feed conversion ratio (FCR).

### White blood cell count and phagocytic assay

Leukocytes were counted in a haemocytometer using Fried-Lukacova solution (475 µL of solution plus 25 µL of blood). White blood cell count determination was done on blood smears stained with Hemacolor (Merck, Darmstad, Germany) by light microscopy. Total numbers of different subtypes of white blood cells was calculated by the formula: total leukocyte count x proportion of differential cells counted (%)/100=absolute levels in g·L^-1^ (Levkut et al., 2009).

### Phagocytic assay

The function of polymorphonuclear cells, monocytes and phagocytic index was assessed by flow cytometry (FACScan, BD Germany) using whole heparinised whole blood and a commercial PHAGOTEST kit (Phagotest, ORPEGEN Pharma, Heidelberg,Germany). The Phagotest kit contains fluorescein (FITC)-labelled opsonised bacteria (*Escherichia coli*- FITC), and reagents to measure the overall percentage of granulocytes and monocytes which ingest one or more bacteria per cell. Cells were analysed by flow cytometry using blue-green excitation light (488 nm argon-ion laser and CellQuest Software). Evaluation of phagocytic cells is described by Čechová et al. (2023). Procedure of staining and measuring was specified in the company protocol.

### Flow cytometry

Jejunal lymphocytes (intraepithelial lymphocytes-IEL, lamina propria lymphocytes-LPL) were isolated and purified by the modified method of SolanoAguilar et al. (2000). Briefly, removed jejunum was placed into an ice-cold buffered Hanks solution (HBSS, pH 7.2 − 7.3), cut longitudinally and lengthwise into 0.5 cm pieces followed by washing three times. The intestine was placed into 50 mL conical plastic tubes (Falcon, BD, Germany) containing warmed (37°C) 5 mM dithiotreitol (HSS-DTT) for removing of mucin in thermostat during 15 min. The supernatant was discarded and gut fragments were rinsed twice in the cold HBSS, followed by incubation of fragments in warmed (37°C) 0.1 mM EDTA-HBSS for 1 h. EDTA released IEL into harvested supernatant. Then the intestine was incubated with 30 mL RPMI-1640 (Sigma, Germany) to remove previous medium during 15 min at 37°C in thermostat. The supernatant was discarded and the gut fragments were incubated in RPMI-1640 with collagenase type I (15 mg/60 mL RPMI; Sigma-Aldrich Chemie Gmbh, Steinheim, Germany) for 1 h at 37°C. Collagenase released LPL into medium. Five minutes shaking intervals was used during all incubations. The harvested supernatants with IEL and LPL were filtered and immediately centrifuged at 600 g for 10 min followed by twice rinse at 250 g for 10 min after resuspention with PBS (Bucková and Revajova, 2014). Isolated lymphocytes were stored in PBS at 4°C to the immunophenotypization and measuring by flow cytometry. Mouse anti-chicken monoclonal antibodies labelled with FITC (Southern Biotech, Birmingham, AL, USA; Cat. No.: CD3 8200-02; CD4 8210-02; CD8 8390-02; IgA 833002; IgM 8310-02;.CD45 8270-02) were used for immunophenotyping of lymphocytes by direct immunofluorescent method. The control antibody, polyclonal goat-anti mouse FITC-conjugated immunoglobulin F(ab’)_2_ fragment (Dako, Denmark) was used at a working dilution of 1:50 with phosphate buffered saline (PBS). After 15 min incubation of cells in dark at room temperature the rince in PBS and centrifugation 5 min at 110 g followed. The lymphocytes were resuspended in 0.2 mL of PBS with 0.1 % paraformaldehyde and storage at 4°C to the measurement by flow cytometry. Procedure for flow cytometry was previously described by Levkut et al. (2019).

### Collection of jejunum samples for ELISA

During necropsy, jejunal segments were taken from the intestine at the same site in each chicken. Length of intestinal segments reached approximately 3 cm. Small pieces of intestinal loops were washed and prepared for determination of sIgA content as well MUC-2 production and secretion. Syringes were filled with an optimal volume (5 mL per each sample) of warm flushing solution (1 M tris/glycine buffer with 0.25 % Tween 20, pH 7; Sigma-Aldrich). Then, a needle was inserted into one end of each intestinal loop, and by emptying the syringe in several pulses, the whole intestinal content was flushed out. The complete luminal brush-lined epithelial wall was flushed, and the content was emptied into 20-mL-volume test tubes. The jejunal flushes were centrifuged for 5 min at 12,000 g (Hettich Rotina 75 420R Centrifuge DJB Labcare, UK) and the supernatants from each sample were used for ELISA (Husáková et al., 2015).

### Detection of sIgA by ELISA

To determine sIgA content in the jejunal flushes, a chicken IgA ELISA kit (Kamiya Biomedical Company) was used. A 96-well microtiter plate was coated with affinity purified anti-chicken IgA antibody. Under laboratory conditions, the volume on each microtiter plate was incubated (22°C, 20 min), and subsequently, the content was aspirated and washed 3 times with solution, following the ELISA kit instructions. Determination of sIgA content was previously described by Levkut et al. (2022).

### Determination of total MUC-2 by ELISA

For detection and determination of total MUC-2, a chicken MUC-2 ELISA kit (Kamiya Biomedical Company) was used. For detection, 96-well microtiter plates were coated with affinity purified anti-chicken MUC-2 antibody. The plates were incubated, then washed and filled with 50 mL substrate solution in each well. The detected samples were diluted 1:5 in PBS with pH between 7.0 and 7.2 and added in 100-mL doses into predesignated wells in duplicates. Mixtures of balance solution in 10 mL and 50 mL of conjugate bound with horseradish peroxidase in stabilizing buffer were added into the plate wells, then incubated at 37°C for 1 h. Determination of total MUC-2 was previously described by Levkut et al. (2022).

### Relative expression of target genes in quantitative real-time PCR (qRT-PCR)

The mRNA levels of occludin and claudin-1 genes were determined in samples of jejunum. Also, mRNA relative expression of the reference gene, coding GAPDH (glyceraldehyde-3-phosphate dehydrogenase). The primer sequences for each primer used for qRT-PCR are listed in Table 2. All primer sets allowed cDNA amplification efficiencies between 94 % and 100 %.

**Table 2:** List of primers used for the chicken gene mRNA quantification.

**Primer**	**Sequence 5′–3′**	**References**
Occludin Fw	ACGGCAGCACCTACCTCAA	Gharib-Naseri et al., 2021
Occludin Rev	GGGCGAAGAAGCAGATGAG	
Claudin-1 Fw	CTTCATCATTGCAGGTCTGTCAG	
Claudin-1 Rev	AAATCTGGTGTTAACGGGTGTG	
GAPDH Fw	CCTGCATCTGCCCATTT	De Boever et al., 2008
GAPDH Rev	GGCACGCCATCACTATC	

Amplification and detection of target products were performed using the LightCycler 480 II Instrument (Roche, Basel, Switzerland) and SsoAdvanced Universal SYBR Green Supermix (Bio-rad, Hercules, California, USA). Subsequent qReal-Time PCR to detect relative expression of mRNA of selected genes was performed for 38 cycles under the following conditions: initial denaturation at 95°C for 2 min, subsequent denaturation at 95°C for 15 s, annealing 60°C for 30 s and final extension step for 2 min at 72°C. A melting curve from 50°C to 95°C with readings at every 0.5°C was generated for each individual qReal-Time PCR plate. All reactions were done in duplicate. We also confirmed that the efficiency of amplification for each selected gene was essentially 100 % in the exponential phase of the reaction, where the quantification cycle (Cq) was calculated. The Cq values of the studied genes were normalised to an average Cq value of the reference gene (ΔCq), and the relative expression of each gene was calculated mathematically as 2^–ΔCq^.

### Histology and morphometry of jejunum

Routine histological method with haematoxylin-eosin staining was used. The height and surface area of the villi in jejunal samples from five chickens of each group (four groups) were analysed. The histological samples were microphotographed (Nikon LABOPHOT 2 with a camera adapter DS Camera Control Unit DS-U 2) and the NIS-Elements version 3.0 software (Laboratory Imaging, Prague, Czech Republic) was used. The height of the villi was measured from the basal region, which corresponded to the higher section of the crypts, the apex (µm). The total cutting surface area of separate intestinal segments included the length and breadth of villi (µm^2^). The data were finally exported to MS Excel and subsequently statistically analysed.

### Statistical analysis

Statistical analysis was performed using GraphPad Prism version 8.3 (GraphPad Software Inc., San Diego, California, USA). The normality of data distribution was assessed using the Shapiro–Wilk test. For comparisons between two independent groups, a two-tailed unpaired Student’s t-test was applied. Data were expressed as the mean ± standard error of the mean (SEM). Differences were considered statistically significant at ^ab/*^P *<* 0.05; ^cd/**^P *<* 0.01; ^ef/***^P *<* *0.001*."

## Results

In previously presented results of our study (Karaffová et al., 2022), it was found that supplementation with *Enterococcus faecium* AL41 promotes β- and γ-globulin fractions and reduces the total number of enterococci in the cecum and feces.

In the second part, the results of the same trial, preformed by growth performance, leukocytic responses in the peripheral blood and intestine, production of intestinal MUC-2 and sIgA, and finally tight juncion proteins are presented. Supplementation with *Enterococcus faecium* AL41 did not affect average daily feed intake (ADFI) and final body weight of broilers on days 8 and 11 of sampling (Table 3). However, average daily body weight gain (ADG) and feed conversion ratio (FCR) were significantly increased in the EF group compared to the control group on day 11 (*P* < 0.05).

**Table 3:** Growth performance parameters.

Parameter	Sampling days	C group ± SEM	EF group ± SEM
Initial body weight (g/bird)	0.	42.8 ± 3.045	41.7 ± 3.098
Final body weight (g/bird)	8. 11.	226.5 ± 13.65 252.3 ± 12.05	229.6 ± 14.00 258.2 ± 13.87
BWG (g/bird)	8. 11.	183.5 ± 4.018 209.3 ± 3.536	186.8 ± 4.165 215.4 ± 4.179
ADG (g/bird/day)	8. 11.	22.94 ± 0.976 **19.03±0.544**	23.35 ± 0.561 19.59 ± 0.092*
ADFI (g/bird/day)	8. 11.	30.34 ± 15.42 38.10 ± 19.41	31.63 ± 17.18 41.24 ± 22.24
FCR (g feed/d of gain)	8. 11.	1.33 ± 0.124 **2.00±0.021**	1.36 ± 0.011 2.11±0.021*

Abbreviations: BWG, body weight gain; ADG, average daily gain; ADFI, average daily feed intake; FCR, feed-to-gain ratio.

Results are presented as mean ± SEM.

In the same row, values with * mean significant difference (*P<0.05*).

White blood cell numbers showed that inclusion of EF did not lead to the significant change in absolute count of total leukocytes, lymphocytes, heterophils, eosinophils, and monocytes in chickens administered EF *per os* (Table 4). Similarly. phagocytic activity, index of phagocytic activity, metabolic activity (Table 5) and index of metabolic activity were not changed (Table 6). Subpopulations of intraepithelial lymphocytes in j*ejunum* as CD3^+^, CD4^+^, CD8^+^, IgM^+^, IgA^+^, and CD45^+^did not show significant shifting (Table 7). However, evaluation of *lamina propria* lymphocytes in *jejunum* (Table 8) measured by flow cytometry showed increase of CD3^+^ lymphocytes at day 5 and 11 in EF group of chickens (*P* *<* *0.001*) comparing to control chickens. Significant increase demonstrated also cells CD4^+^ and CD8^+^ cells at day 8 in EF group (*P* *<* *0.001*) compare to control. Number of IgA^+^ cells was higher at day 8, 11 (*P* *<* *0.001*) in EF group than in control group. Finally, CD45^+^showed increase of at day 11 (*P* *<* *0.001*) compare to control chickens.

**Table 4:** White blood cell counts in peripheral blood (absolute number – G.L^-1^ mean ± SEM).

Parameter	Sampling days	Control group ± SEM	EF group ± SEM
Leukocytes	5. 8. 11.	8.56±2.05 5.66±0.8 7.47±1.94	8.99±1.45 6.02±1.46 7.76±1.76
Lymphocytes	5. 8. 11.	5,00±1.44 3.76±0.49 4.99±1.33	4.98±0.7 4.37±1.16 5.29±1.13
Heterophils	5. 8. 11.	3.22±0.78 1.68±0.62 2.12±0.7	3.7 ± 1.19 1.34±0.53 2.12±0.8
Eosinophils	5. 8. 11.	0.14±0.06 0.09±0.04 0.17±0.09	0.14±0.05 0.1 ± 0.05 0.15±0.07
Monocytes	5. 8. 11.	0.22±0.07 0.15±0.06 0.19±0.1	0.19±0.08 0.16±0.1 0.2 ± 0.1

**Table 5:** Phagocytic activity (FA) and index of phagocytic activity (IFA).

Parameter	Sampling days	Control group ± SEM	EF group ± SEM
FA	5. 8. 11.	60.23±4.92 26.2 ± 8.87 36.27±7.81	43.17±9.13 26.37±8.35 23.23±10.09
IFA	5. 8. 11.	262±11.0 1285±324 777,0 ± 146	242±5.0 1446±228 729±57.0

**Table 6:** Metabolic activity (MA) and index of metabolic activity (IMA).

Parameter	Sampling days	Control group ± SEM	EF group ± SEM
MA	5. 8. 11.	35.05±6.55 89.36±3.55 28.11±4.64	46.96±8.98 90.87±6.02 28.74±9.93
IMA	5. 8. 11.	411±82.5 1285±202 311±106	532±127 1239±172 222±19.3

**Table 7:** Subpopulations of intraepithelial lymphocytes (IEL) in the jejunum (relative percentage; mean ± SEM).

Subpopulation	Sampling days	Control group ± SEM	EF group ± SEM
CD3	5. 8. 11.	16.74±3.81 11.39±5.38 28.99±18.19	24.39±7.52 16.64±4.24 44.62±8.32
CD4	5. 8. 11.	9.31±2.57 11.54±8.09 20.79±12.95	14.3 ± 7.42 12.29±5.91 27.45±3.9
CD8	5. 8. 11.	28.72±7.09 16.18±8.8 31.95±10.28	33.53±13.61 16.69±4.71 42.16±8.36
IgM	5. 8. 11.	20.34±5.96 11.69±4.41 33.92±12.48	24.45±8.17 11.64±3.18 40.68±13.78
IgA	5. 8. 11.	6.61±2.92 9.3 ± 5.9 20.63±12.89	13.81±6.49 12.29±6.47 33.49±9.72
CD45	5. 8. 11.	92.7 ± 0.82 82.28±3.96 87.19±3.18	92.8 ± 2.67 80.23±1.96 86.42±5.73

**Table 8:** Subpopulations of lamina propria lymphocytes (LPL) in the jejunum (relative percentage; mean ± SEM).

Subpopulation	Sampling days	Control group ± SEM	EF group ± SEM
CD3	5. 8. 11.	**11.16±0.86** **13.51±0.75** 19.6 ± 4.47	**17.82±1.55**^⁎⁎^ **21.9±1.04**^⁎⁎^ 29.01±6.73
CD4	5. 8. 11.	9.99±2.04 **11.98±0.58** 8.08±0.95	13.46±5.49 **19.54±0.69**^⁎⁎^ 11.84±4.31
CD8	5. 8. 11.	9.76±4.15 **10.95±0.91** 15.67±5.92	13.03±5.06 **19.6±1.24**^⁎⁎^ 21.9 ± 12.55
IgM	5. 8. 11.	11.58±2.03 14.01±1.49 12.39±13.83	12.92±3.79 17.38±3.0 14.81±5.39
IgA	5. 8. 11.	11.88±5.22 **10.79±0.85** 13.00±1.02	12.2 ± 3.03 **18.9±1.41**^⁎⁎^ 22.6 ± 1.65ac
CD45	5. 8. 11.	15.68±8.58 84.6 ± 5.49 **55.4±4.52**	17.17±4.29 84.19±4.14 **71.23±5.44**^⁎⁎^

Results are presented as mean ± SEM.

In the same row, values with ^⁎⁎^ mean significant difference (*P<0.01*).

**Fig. 1:**
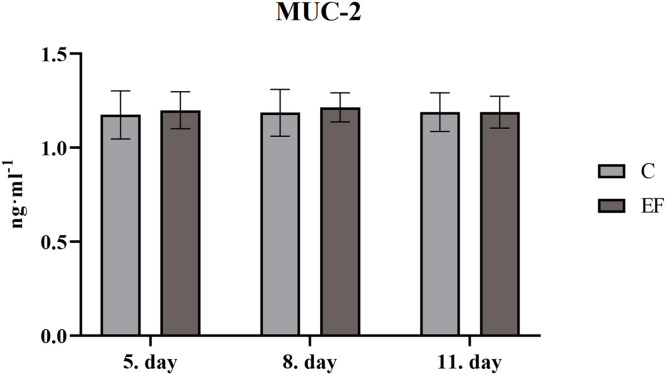
Concentration of MUC-2 (ng·mL^-1^) in jejunum.

**Fig. 2:**
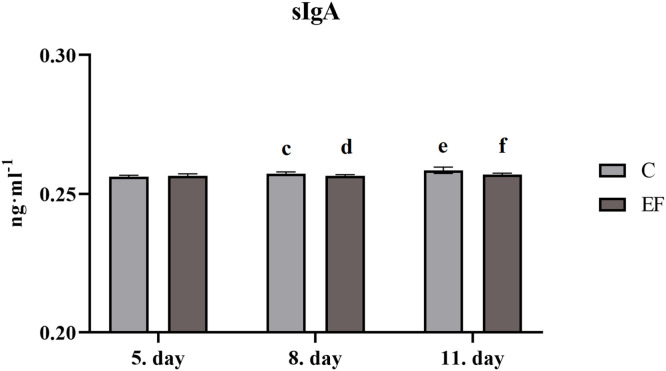
Concentration of sIgA (ng·mL^-1^) in jejunum, Legend: superscripts indicate significant differences between control and experimental group: *^cd^P<0.01; ^ef^P<0.001.*

Concentration of MUC-2 in intestinal flush at day 5, 8 and 11 of age was not changed in *jejunum* of chickens with *Enterococcus faecium* AL41 comparing with those fed control diet (Fig. 1). However, quantity of IgA in intestinal flush was lower at day 8 (*P* *<* *0.05*) and 11 (*P* *<* *0.001*) than in control group (Fig. 2).

**Fig. 3:**
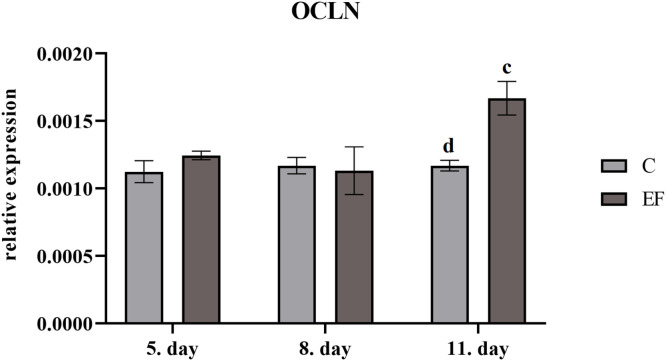
Relative expression of occludin gene in quantitative real-time PCR**,** Legend: superscripts indicate significant differences between control and experimental group: *^cd^P<0.01.*

**Fig. 4:**
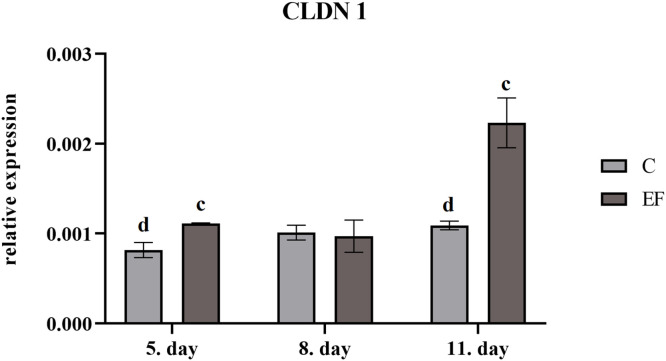
Relative expression of claudin 1 gene in quantitative real-time PCR, Legend: superscripts indicate significant differences between control and experimental group: *^cd^P < 0.01.*

Relative expression of occludin gene was higher at day 11 in EF (*P* *<* *0.05*) than in control chickens (Fig. 3). Similarly, relative expession of claudin1 gene was upregulated in EF at day 5 (*P* *<* *0.01*) and day 11 (*P* *<* *0.01*) comparing to control chickens (Fig. 4).

Height of villi showed increase in EF (*P* *<* *0.001*) comparing to those fed control diet at day 11 of age (Table 9). On the other hand, cutting surface of villi increased in EF at day 5 (*P* *<* *0.001*) and 11 (*P* *<* *0.05*) of age compare to control group. Data obtained from depth of crypts showed their decrease (*P* *<* *0.001*) at day 5 in EF supplemented chicks. However, reverse increase of depth of crypts was found at day 8 (*P* *<* *0.001*), and 11 of age (*P* *<* *0.05*) in EF group (Table 9).

**Table 9:** Morphometrical parameter analysed in the intestinal mucosal villi of jejunum.

Sampling day	5.		8.		11.	
Group	***C*** ± SEM	**EF** ±SEM	***C*** ± SEM	**EF** ± SEM	***C*** ± SEM	EF ± SEM
Height of villi (µm)	531.55± 84.2	518.67± 99.8	689.16± 158.5	694.74± 113.4	**784.21± 154.65**	911.88± 179.62***
Cutting surface of villi (µm^2^)	**41685.5±** **151381**	**59701.1±** 2745.3***	72220.06± 30140.47	71145.95± 22558.29	**82811.3±** **36280.86**	**70109.92±** 20834.86*
Depth of crypts (µm)	**109.74± 25.45**	100.9±22.16***	**90.05± 24.78**	102.59± 25.64***	**108.5±27.35**	119.35± 33.46*

Results are presented as mean ± SEM.

In the same day, values (between C and EF group) with * mean significant difference *P<0.05;* *** mean significant difference *P<0.001.*

## Discussion

The interaction between the intestinal immune system and commensal microbiota in chicks begins at hatching and the avian immune system concurrently reacts to changes in the luminal microenvironment during growing of broilers. The intestinal mucosa represents the most active defence barrier against the continuous challenge of food antigens and pathogenic microorganisms invading the intestinal lumen (McGuckin et al., 2011).Previosly, has been demonstrated that a supplementation of the chicken diet by either prebiotic, probiotic or synbiotic has an impact on barrier function (Markowiak and Śliżewska, 2018; Selecká et al., 2021; Shehata et al., 2022). Our previous studies showed that modification of gut microbiota by dietary *Enterococcus faecium* improved muscle health and meat quality (Žitňan et al., 2019; Albrecht et al., 2022), activated immune response of intestine in broilers, as shown by an upregulation of mucin (e.g. MUC-2) as well as selected cytokines (Revajová et al., 2022; Karaffová et al., 2022).

In the current data of our trial dietary supplementation of EF increased average daily body weight gain on day 11. Similarly, on that day feed conversion ratio increased in experimental group. These results are consistent with previously presented data (Albrecht et al., 2022) concerning increased weight of pectoralis major muscle in chickens administrated EF *per os* for seven days. Interestingly, our data were obtained in brooding period of feeding. Albrecht et al. (2022) indicate effect of capillarization and tissue perfusion in EF treated chicks, that manifests at day 8 and/or day 12 of age which is in agreement with our results for 11day-old EF-supplemented chicks.

The basic protection of the mucous membranes is mediated by mucin produced by goblet cells, which is either localized on the cell membrane or secreted into the lumen to form a mucosal layer. In chickens, secretory MUC-2 is a major constituent of mucins in the small and large intestinal mucus layer (Zhang et al., 2015). In the current experiment chickens fed diet *Enterococcus faecium* AL41 did not alter level of MUC-2 in followed segment of the intestine. The influence of *E. faecium* EF55 on the dynamics of intestinal mucin production in birds infected with *Salmonella* Enteritidis was previously demonstrated by Levkut et al. (2012). In our experiment chicks fed diet supplemented *E. faecium* AL41 were intact without clinical changes. Our recent study (Karaffová et al., 2022) showed, that administration of *E. faecium* AL41 upregulated gene expression for MUC-2 and IgA mainly on day 11 of age, which may indicate a cumulative effect of *E. faecium* AL41 on selected genes of non-specific mucosal immunity in groups with EF supplementation.

Secretory IgA produced by IgA^+^ plasma cells is transported to the lumen of the mucosal layer by epithelial cell, where it protects the epithelium against colonization by pathogens (Macpherson et al., 2008). Lack of IgA expression during the first week post-hatch in birds has been explained by the presence of maternal IgA from the egg and their role in passive immune mechanism (Bar-Shira et al., 2014). In current experiment, data showed that concentration of sIgA after prolonged feeding of diet with *E. faecium* AL41 was decreased in jejunum of chicks. Recently, results reported from this experiment (Karaffová et al., 2022) has shown that administration of *E. faecium* AL41 resulted in significant reduction of the total number of enterococci in the cecum and faeces of broilers. It can be hypothesized that positive microbial changes in the intestinal tract resulted in decreased quantity of sIgA in intestinal flush. This is supported also by fact that, there was no infection, and the broilers remained in a good condition. In any case, there are only a few studies about the influence of *E. faecium* alone on the parameters of mucosal immunity in chickens .

Previously, *E. faecium* AL41 revealed the protective effect and positive influence on the local and systemic immune response in *Salmonella* Enteritidis PT4 infected chickens. Cecal IEL and LPL lymphocytes in that experiment showed at 7 dpi stimulation of CD3^+^, CD4^+^, and CD8^+^ subpopulations in probiotic groups, especially in EFSE group (Revajová et al., 2022). In current experiment chickens fed *E. faecium* AL41showed stimulation only of LPL CD3^+^ at 5 dpi, CD3^+^, CD4^+^, CD8^+^, and IgA^+^ cells at 8 day dpi, and IgA^+^cells and CD45^+^ lymphocytes at day 11. It is known that CD45 is an antigen found on the surface of all nucleated hematopoietic cells (Shivtiel et al., 2008;Wellhausen et al., 2023) what only confirm stimulation of followed lymphocytes.

It is supposed that activation of lymphocytes can be induced by prolong administration of used probiotic bacteria. In this study, adding of *E. faecium* AL 41 to broilers did not show any impact on white blood cells counts, bacterial metabolic activity, and phagocytic activity. In addition, the applied probiotic product did not affect number of immunocompetent cells in intraepithelial layer of the current birds. Preventive effect of EFAL41demonstrated Revajová et al. (2022) in *salmonella* infected broilers. The authors found higher density of IgA^+^intraepithelial lymphocytes and IgA^+^ and IgM^+^ *lamina propria* lymphocytes in group EFAL41 infected SE PT4.

One of the major components of the intestinal barrier is the formation of tight junction (TJ) between epithelial cells. In current study followed claudin1 and occludin were upregulated in chickens fed *E. faecium* AL41. Proszkowiec-Weglarz et al. (2020) in their study followed expression of tight junction proteins including occludin and claudin1 in the jejunum and ileum of broilers. The level of these proteins decreased after hatch and became stable during the first 2 weeks of life. The authors supposed that expression of tight junction proteins could be a result of compensatory mechanism responding to alterations in microbial composition and restoring intestinal permeability. Some bacterial pathogens can impair intestinal barrier function by disruption of tight junctions and initiation of inflammatory cascades (Guttman and Finlay, 2009; Shifflett et al., 2005). In this regard, Berkes et al. (2003) reported that many enteropathogenic bacteria have been implicated in the disruption of tight junctions including enteropathogenic *Escherichia coli, Clostridium difficile, Clostridium perfringens, Helicobacter pylori, Campylobacter jejuni, Campylobacter concisus*, and *Salmonella* Typhimurium. Tight junction proteins also play important roles in signal transduction mechanisms that regulate cell proliferation, differentiation and gene expression (McCrea et al., 2009). Several studies showed that modification of gut microbiota by dietary *Bacillus* spp. improved gut barrier integrity and activated immune response in the ileum of broilers, as shown by upregulation of tight junction proteins as occludin, ZO-1 (zonula occludens-1), and JAM-2 (junctional adhesion molecule-2) (Lee et al., 2015; Bilal et al., 2021;Duangnumsawang, 2023; Cai et al., 2024). However, there are no studies comparing the influence of *Enterococcus faecium* on tight junction proteins.

The jejunum with its crypt-villus morphology is the primary site of global nutrient absorption. The surface area of the small intestine is increased many-fold due to the presence of villi, while its structure could be simultaneously affected by commensal or pathogenic microorganisms, toxins residing in the gut (Awad et al., 2006). In current experiment administration of *E. faecium* AL41 individually *per os* to chickens induced improved height of villi, width of cutting surface of villi, and depth of crypt in jejunum of chickens fed feeding with *E. faecium* AL41. The observed changes in gut morphology signify an enhanced absorptive surface area and capacity. Gut epithelial cells are proliferated and gradually differentiated by crypts of Lieberkühn glands (Xiang et al., 2023). Increase of depth of crypts in jejunum of experimental group can be explained by higher density of immunocompetent cells in mucosa at day 8 and 11of the experiment as prolong adimistration of probiotic bacteria. Improvements in intestinal morphology and increase of barrier function following *E. faecium* supplementation can suggests also better growth performance of chickens kept in our trial.

## Conclusion

In conclusion, seven days administration of *E. faecium* AL41 to chicks after hatching, seems to positively influenced intestinal morphology, intestinal barrier integrity and intestinal immunity in broilers. Our results also suggest a growth-promoting effect of the applied probiotic strain in chicks during the early developmental period, which may enhance its potential for further use in intensive poultry production.

## Declaration of compnent interest

The authors have no pertinent financial or non-financial conflicts of interest to declare.
